# Pulse Wave Velocity Estimation in a Controlled In Vitro Vascular Model: Benchmarking Machine Learning Approaches

**DOI:** 10.3390/s26031066

**Published:** 2026-02-06

**Authors:** Daniel Barvik, Martin Černý, Michal Prochazka, Norbert Noury

**Affiliations:** 1 Department of Cybernetics and Biomedical Engineering, VSB-Technical University of Ostrava, 17. Listopadu 2172/15, 708 00 Ostrava, Czech Republic; 2 Biomedical Sensors Group, Institute for Nanotechnology of Lyon, University of Lyon, 69100 Lyon, France

**Keywords:** physical vascular model, pulse wave velocity, regression, adaptive neuro-fuzzy inference system

## Abstract

This study evaluates the feasibility of estimating stiffness-related parameters and pulse wave velocity (PWV) in a controlled in vitro circulatory setup using artificial silicone vessels with systematically varied Shore A hardness and wall thickness. From synchronized pressure and capacitive waveforms, fiducial points and engineered features are extracted, together with pump settings (stroke volume and heart rate). A Sugeno-type adaptive neuro-fuzzy inference system (ANFIS) is used for hardness-level prediction and benchmarked against linear regression and contemporary machine-learning/deep-learning baselines using stratified cross-validation. PWV estimates derived via hardness-to-elasticity conversion models and the Moens–Korteweg formulation are evaluated against a reference PWV obtained within the same experimental configuration. Under these controlled conditions, the proposed pipeline shows strong agreement with reference labels and measurements. The results should be interpreted as an in vitro validation step; translation to biological tissues or in vivo data will require external validation, calibration of material-property mapping, and robustness testing under physiological variability and measurement noise.

## 1. Introduction

Cardiovascular diseases remain a major cause of morbidity and mortality worldwide, and long-term pressure loading can progressively change arterial wall properties and increase arterial stiffness [1]. In this context, pulse wave velocity (PWV) is widely used as a functional marker linked to arterial stiffness, but PWV interpretation depends on vessel mechanics and measurement conditions. Experimental circulatory simulators and vascular phantoms are therefore often used to study wave propagation under controlled settings and to support method development [2,3]. At the same time, such setups are simplified and cannot fully reproduce the complex behavior of the human vascular system.

Systemic circulation is a coupled network of compliant vessels, and pulsatile flow produces periodic loading of the arterial wall. Direct measurement of wall stress or elasticity in vivo remains challenging, and physical or mathematical models are commonly used to understand biomechanical behavior and to interpret measured waveforms [4,5,6]. In vitro models can provide synchronized signals (e.g., pressure and volume pulse waves), together with well-defined material and geometric settings, which can then be used as inputs for data-driven analysis of stiffness-related parameters [7].

Compliance and stiffness are inversely related: as arteries stiffen, compliance decreases [8]. In practice, waveform shape changes with stiffness (e.g., a less pronounced dicrotic notch in stiffer vessels), and such features can be used to characterize controlled phantom conditions. In this study, we use artificial arterial segments with several wall thickness levels and hardness levels to emulate a broad range of stiffness conditions. We apply an adaptive neuro-fuzzy inference system (ANFIS) to model the relationship between experimental conditions, pulse wave features, and stiffness-related targets supporting PWV estimation. Recent studies increasingly adopt data-driven approaches, including machine learning and deep learning, to extract clinically relevant information from pulse-related signals and to enable cuffless cardiovascular monitoring pipelines [9,10,11].

Since ANFIS-based PWV estimation is not a new concept, we also benchmark ANFIS against contemporary machine learning and deep learning baselines on the same dataset to provide a transparent performance context. The main contributions of this work are: an in vitro dataset acquired in a controlled circulatory setup with systematic variation in stroke volume, heart rate, wall thickness, and material hardness; a fuzzy logic-based modeling approach (ANFIS) with clearly defined training and evaluation; and benchmarking against modern ML/DL baselines. In addition, PWV estimates are evaluated against reference measurements obtained in the same experimental setup. The manuscript is organized as follows: Section 2 describes the experimental setup and methods, Section 3 presents the results, and Section 4 discusses limitations and future work.

## 2. Materials and Methods

The dependency of vessel elasticity was investigated using our physical vascular model [6,12,13]. This allows us to test and validate the approach under stable experimental conditions before considering in vivo use. The Windkessel-based setup can be tuned using a Harvard Apparatus pulsatile pump by adjusting heart rate, stroke volume, and pulsatility. Further tuning could be achieved using various types of hoses from different materials and reservoirs [6,13]. Every single parameter change can affect the shape of the pulse wave; thus, we choose the dependence of vessel elasticity on the shape of the pulse wave and its effect on pulse wave velocity using the Moens–Korteweg Equation (1) [14,15,16], due to its widespread use in non-invasive PWV estimation. It provides a direct analytical relationship between PWV, wall elasticity, thickness, and diameter, which is useful for model validation in controlled experimental setups. The limitations of this formulation and the related assumptions are discussed below.

(1) $$PWV=\frac{D}{PTT}=\sqrt{\frac{EW}{\rho d}}$$

where *D* is the distance between the two pressure measurement sites, PTT is the pulse transit time, *E* is the Young’s modulus, *W* is the wall thickness, ρ is the fluid density, and *d* is the inner diameter. The conversion models used to map Shore A hardness to *E* are summarized in Table 1.

The Moens–Korteweg equation, while widely used, relies on several simplifying biomechanical assumptions. It treats the arterial wall as an ideal elastic, homogeneous, isotropic, and thin-walled tube, and assumes the fluid inside is incompressible. In our physical model, we approximate these conditions by using uniformly manufactured silicone tubes with constant diameter and wall thickness, and by ensuring a constant mean pressure throughout the system. However, the viscoelastic behavior of real arteries and non-Newtonian properties of blood are not captured in this setup. As a result, the estimated PWV values are valid within the controlled lab context but may differ in vivo, where arterial wall properties and flow dynamics are more complex. Future work should consider incorporating viscoelastic and fluid–structure interaction models for improved physiological fidelity.

**Figure 1:**
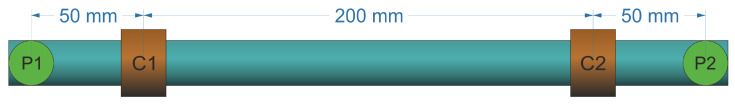
Sensors placement on artificial arterial segment [13].

For our experiment, we used artificial artery segments made from silicone material to obtain such variation in measurements using different properties (Shore A hardness 10–50, and wall thickness 1, 1.5, 2 mm). The pressure and capacitive sensors [13] were used for data acquisition, and were placed on the artificial artery segment in the same places for each measurement setup. The pressure sensors were placed on each end of the artery segment, and the second type of sensor, the capacitive sensor, was placed 5 cm from each end, as shown in Figure 1 [13].

Each measurement corresponds to a fixed combination of pump settings (heart rate and stroke volume) and tube configuration (wall thickness and material hardness). This structure allows for evaluation not only under random splits, but also under more challenging protocols where entire configurations are held out. To reduce optimistic bias in a controlled dataset, we also consider group-based evaluation (e.g., holding out complete tube configurations or complete setting combinations), which better reflects the expected domain shift when moving beyond a single controlled setup.

Data were acquired, preprocessed and synchronized directly on the microcontroller (STM32, ST Microelectronics, Crolles, France). One of the pressure sensors was used as a pressure feedback for a digitally controllable valve to maintain constant mean arterial pressure. All calculations are performed in real time within the microcontroller, and the acquired data, at a sampling rate of 200 samples per second, are transferred to the PC. For the experiment we used stroke volumes of 10 and 20 mL, heart rates of 30, 60, 80 bpm, three mentioned wall thicknesses and five different silicone hardnesses. At least 15 min of data for each combination of artificial arterial segment and pump settings were acquired. In total, 90 measurements were performed. The selected stroke volumes and heart rates were chosen to reflect a range of both physiological and pathological conditions. A stroke volume of 10 mL simulates a weakened cardiac output, which may be present in conditions such as heart failure or severe vascular constriction, while 20 mL reflects a healthy or enhanced cardiac state. Similarly, a heart rate of 30 bpm represents bradycardia (seen in some pathological states or under anesthesia), whereas 60 and 80 bpm correspond to typical resting and slightly elevated heart rates, respectively. These parameters were also limited by the capabilities of the programmable pulsatile pump, which is designed to simulate cardiovascular activity of medium-sized mammals such as monkeys or dogs. This setup enabled observation of the sensitivity of PWV to different hemodynamic loading conditions across both healthy and impaired vascular models.

**Figure 2:**
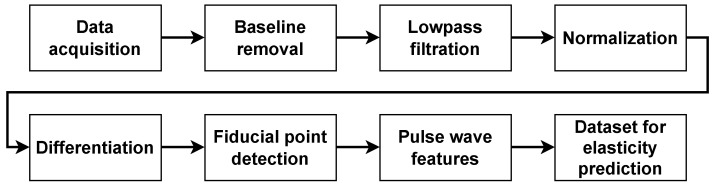
Data processing workflow for PWV estimation.

All measured data were loaded into the Matlab environment for complete signal processing shown in Figure 2.

At the first step, the baseline was removed using a high-pass zero-phase digital filter with a cutoff of frequency 0.2 Hz [19], which removes only the constant component, identified as a maintained pressure of 30 mmHg, which is not significant for future pulse wave analysis. The following low-pass zero-phase digital filter was adaptively selected by Welch’s power spectral density estimation of each signal to ensure a minimum of 4four harmonic frequencies [20], which are the most significant in our analysis. Therefore, we used three cutoff frequencies in total for each pump heart rate value. The cutoff frequencies were 8 Hz, 6 Hz and 3 Hz. The next step for pulse wave analysis was to extract features from each pulse wave by identifying fiducial points on the pulse wave and its derivative, as these points provide important information for further analysis. Fiducial points are key, easily identifiable landmarks on a pulse waveform that correspond to physiologically significant events during the cardiac cycle. These points—such as the pulse foot, systolic peak, and dicrotic notch—serve as temporal anchors for calculating various diagnostic features like pulse transit time, systolic duration, and waveform morphology. Their accurate identification is essential for reliable extraction of cardiovascular parameters, as they directly reflect arterial stiffness, blood ejection, and wave reflection phenomena. In this study, fiducial points were detected automatically using zero-crossing techniques on pulse wave derivatives to support consistent and objective signal analysis. To keep terminology consistent, we use the term foot (pulse onset) to indicate the same waveform landmark throughout the manuscript. For the reference PTT estimation between the two pressure signals, we detect the same fiducial point (foot) in both channels on a beat-to-beat basis. The resulting PTT values are then aggregated per trial (e.g., median across beats) to reduce sensitivity to occasional mis-detections.

The following fiducial points were selected based on the literature search [9,21,22]:

- The foot of the pulse wave, the last minimum before the systolic peak, considered to bre the beginning of the pulse wave;
- The systolic peak, based on blood ejection from the left ventricle (pump in our case), the result of the forward wave traveling the arterial tree and a backward wave returning to the heart from the reflection sites;
- Rising steep, the point of maximum upslope on first derivative;
- The falling steep, first local minimum after maximum upslope on first derivative;
- The dicrotic notch, small dip or notch that appears on the arterial pressure waveform during the cardiac cycle.

**Figure 3:**
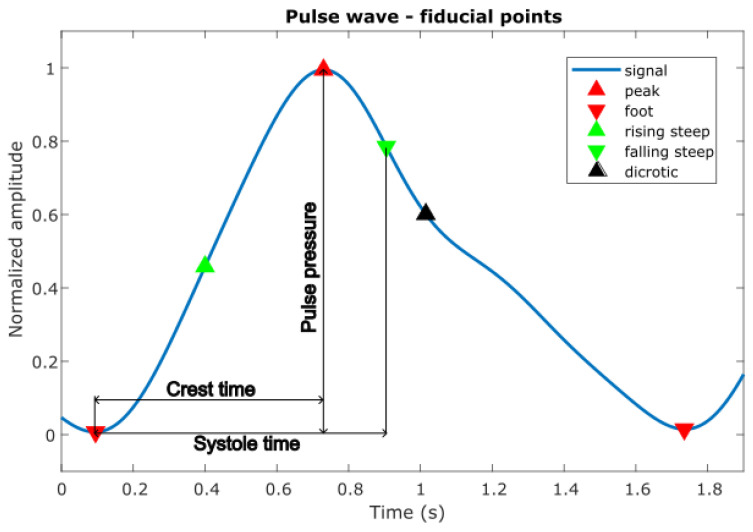
Example of fiducial point detection on pulse waveform.

All mentioned fiducial points (Figure 3) were crucial in understanding the pulse wave and extracting major features for further analysis.

For each trial, multiple cardiac cycles were recorded. Fiducial points and derived features were computed per beat and then aggregated to obtain one feature vector per trial used for model training and evaluation.

From fiducial points we can calculate other features described in [19], as shown on Figure 3.

- Pulse-to-pulse interval, characterizes the stability of our vascular model;
- Crest time, time difference between the pulse onset and the first zero-crossing of the pulse wave derivative;
- Systole time, time difference between foot and falling steepest point;
- Pulse pressure, amplitude between foot and systolic peak of pulse wave.

Acquired data were normalized to an amplitude range from 0 to 1, and fiducial points were automatically detected by a zero-crossing detector applied on the first, second, and third derivatives [19,23,24]. Zero-crossing is the current method used to localize slope change points, but it needed to be tuned to detect certain fiducial points.

Afterwards, we calculated the pulse pressure from the data before amplitude normalization and calculated the crest time and the systole time. The influence of these features was tested for statistically significant relationships between tunable parameters and calculated features.

### 2.1. Machine Learning and Deep Learning Baselines

To provide an up-to-date performance context beyond linear regression and ANFIS, we implemented a benchmark of contemporary machine learning (ML) and deep learning (DL) models for hardness-level prediction [7,25]. The target was treated as discrete Shore A classes (FULL: {10, 20, 30, 40, 50}; LOW: {10, 20, 30}). All models used the same feature set derived from the fiducial-point analysis and pump/tube settings.

### 2.2. Feature Set and Feature Engineering

The baseline input features were: stroke volume (SV), heart rate (HR), wall thickness (W), crest time, pulse pressure (PP), and systole time. In addition, we evaluated an engineered feature set (FE) containing physiologically motivated ratios and normalized timing features. Specifically, we computed the RR interval as RR≈60/HR, normalized timing features (e.g., systole time and rise time relative to RR), compliance and stiffness proxies (e.g., SV/PP and PP/SV), interactions with wall thickness (e.g., PP/W and W·PP), and log-transformed variables (e.g., log(1+PP) and log(1+SV)). These operations were designed to stabilize variance and capture nonlinear relationships in a small dataset.

### 2.3. Cross-Validation Protocol and Metrics

Model evaluation was performed using stratified 5-fold cross-validation to preserve the class distribution in each fold. For performance reporting we used macro-averaged F1-score as the primary metric, together with macro-averaged sensitivity and specificity (one-vs-rest), which are more informative than accuracy in small, multi-class settings. Random seeds were fixed to ensure reproducibility.

### 2.4. Model Families

The ML benchmark included: linear classifiers (logistic regression) and nonlinear margin-based models (SVC with RBF kernel), instance-based learning (k-nearest neighbors), and tree-based ensembles (random forest and histogram-based gradient boosting). In addition, we evaluated regression models whose continuous predictions were snapped to the nearest discrete Shore A class (ridge, polynomial ridge, polynomial lasso, SVR with RBF kernel, random forest regressor, and gradient boosting regressor).

The DL benchmark included three small neural-network baselines designed for small-data learning: a standard MLP with softmax output, an ordinal CORAL formulation to exploit the ordered nature of Shore A levels, and an MLP with Monte-Carlo dropout used at inference time to estimate predictive uncertainty. For DL models, features were standardized within each fold and early stopping was applied using an inner stratified split of the training fold.

Further work for hardness estimation had two directions, linear regression and fuzzy system; we used the same input data in both methods.

**Figure 4:**
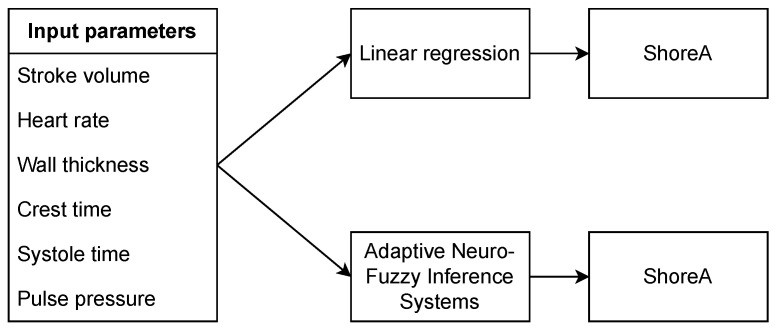
Two models for Shore A hardness estimation based on input parameters.

We fitted the regression model for hardness using pulse wave features and other directly measurable parameters from our experiment setting as predictors, as shown in Figure 4.

In this study, we use an adaptive neuro-fuzzy inference system (ANFIS) [26], which combines fuzzy logic with neural networks, as an interpretable model for stiffness-related target estimation within a controlled in-vitro dataset. The goal is to evaluate whether ANFIS can capture nonlinear relationships between experimental settings, pulse wave features, and hardness-related targets that support PWV-related analysis under laboratory conditions.

ANFIS was selected because it provides a rule-based structure and can model nonlinear relationships with a relatively small dataset [27]. In this work, ANFIS is evaluated alongside contemporary ML/DL baselines to provide a transparent performance context under the same controlled experimental conditions.

To reduce overfitting in Matlab, the dataset was divided into training (75%) and testing (25%) subsets, so that the model was evaluated on previously unseen data. Model complexity was controlled by limiting the number of membership functions and the number of training epochs. Where applicable, early stopping was used based on validation performance. The reported results therefore reflect performance within this controlled in-vitro dataset; stronger external-style validation (e.g., group-based testing) is planned to better assess generalization across configurations.

We implemented a Sugeno-type ANFIS with six inputs and one output. Configuration is mentioned in Table 2. Membership functions were selected as Gaussian functions, and the initial rule base was created using a data-driven procedure to avoid manual tuning. Training was performed using the standard hybrid learning algorithm (least-squares estimation for consequent parameters and gradient-based updates for premise parameters). Fixed random seeds were used where applicable to ensure reproducibility.

The number of epochs was set to 30, and the input data order was randomized to reduce learning of input patterns caused by the ordering of samples and file names.

The architecture of ANFIS consists of five layers [26,28,29,30]:

1. Input layer—six input variables represented by membership functions;
2. Fuzzy inference layer—generates the fuzzy rules;
3. Normalization layer—computes the weights of each rule;
4. Defuzzy layer—combines all rules to generate single output variable;
5. Output layer—final output of the ANFIS model.

**Figure 5:**
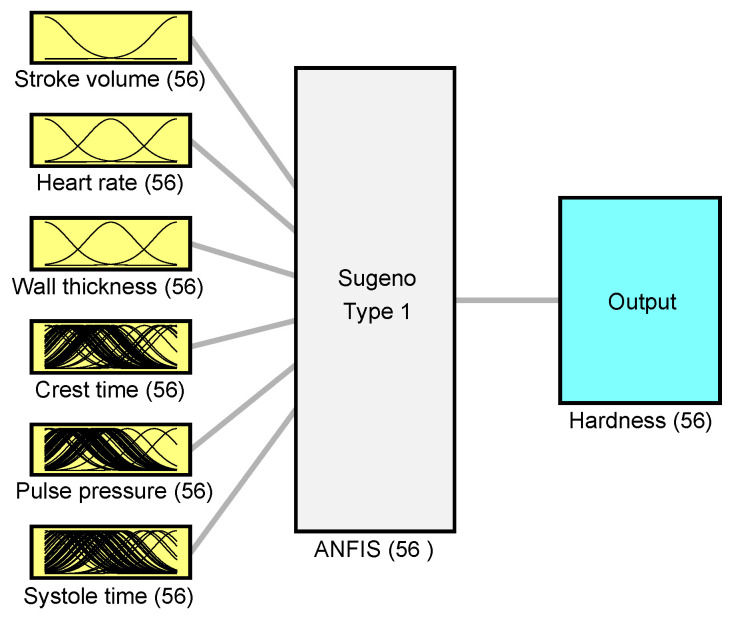
ANFIS model description for input and output membership functions.

The ANFIS was trained by a hybrid learning algorithm using backpropagation and least-squares estimation. Least-squares estimation was used to update parameters in layers 2 and 3 and backpropagation was used in layers 4 and 5 to update the parameters of the neural network, as shown in Figure 5.

Beyond correlation-based metrics, we report error-based metrics (MAE and RMSE) for PWV-related estimates and summarize errors across experimental conditions (e.g., by hardness level and wall thickness) to show where the model performs reliably and where it tends to fail. In addition to the reported stratified cross-validation, future work will include group-based protocols where entire tube configurations are held out during testing (e.g., leave-one-configuration-out) to quantify domain shift.

Hardness-related modeling was evaluated using metrics consistent with the target formulation: $R^{2}$ for regression-style outputs, and macro-averaged F1-score (with sensitivity/specificity) for discrete hardness-level classification in the ML/DL baselines. These models were further used for estimation of the pulse wave velocity based on conversion of the hardness value to elasticity described by five models found in the literature review [17,18,31,32,33]. The elasticity values were fitted in Moens–Korteweg Equation (1) to obtain estimated PWV.

To assess predictive performance, we report both classification and error-based metrics. Classification performance is quantified using macro-averaged F1-score, sensitivity (recall), and specificity (one-vs-rest). In addition, to enable an interpretable error-based comparison across heterogeneous ML/DL predictors, we report the mean absolute error (MAE) and root mean squared error (RMSE) computed on numeric Shore A values. For classification-based models, predicted class labels (e.g., 10/20/30/40/50) were directly treated as numeric Shore A values when computing MAE and RMSE.

Different hardness-to-elasticity conversion models can yield different estimates of Young’s modulus for the same artificial vessel, which propagates into different model-based PWV estimates through Equation (1). For a given tube, PWV is physically single-valued; here we compare the resulting model-based PWV values (using the alternative conversions, as well as hardness predicted by regression/ANFIS) against the reference PWV measured in our experimental setup to quantify sensitivity to the conversion mapping.

The last step of our experiment was the verification measurement of pulse wave velocity on our physical vascular model with a higher sample rate. We used pressure sensors only, because the capacitive measurement is limited to only 200 samples per second. With the pressure sensors we could reach a sample rate of 2000 samples per second, which was sufficient to measure pulse wave delay between two pressure sensors with a known distance of 30 cm. The measured values of PWV were compared with the estimated values from our models. In the first comparison, we calculated the difference between the estimated PWV values from the regression and fuzzy models and those of the theoretical calculation. Further comparison was done on the difference between the regression, fuzzy and theoretical PWV values and the measured PWV values. Thus we can validate our prediction models to observable pulse wave velocity. This predicted PWV values were statistically tested for normality using the Shapiro–Wilk normality test and for significant difference in mean error from 0 using Student’s *t*-test. To facilitate description we have used subscripts based on the method used: $PWV_{R}$ indicates values obtained by the regression method, $PWV_{F}$ indicates the fuzzy method, $PWV_{T}$ stands for theoretical calculation and $PWV_{M}$ is based on real measured values. In this manuscript, “single-site” refers to the fact that the model inputs can be derived from the waveform measured at one location (together with known pump settings and tube geometry). For validation, PWV is obtained from a two-sensor reference measurement within the same setup by estimating the pulse transit time (PTT) between the upstream and downstream pressure signals, and using PWV=D/PTT, where *D* is the fixed distance between the sensors measured along the tube axis. Therefore, the present study is framed as an in vitro feasibility and validation step under controlled conditions, not as proof of clinical performance.

## 3. Results

Our physical vascular model allowed for continuous tuning of the pump parameters; however, for this study we used only discrete parameter values. A key limitation was the use of a limited set of artificial arterial segments, which could only represent stiffness variations in discrete steps. A quantitative comparison between ANFIS and the linear regression baseline is reported in Section 3.2 (Table 4), including $R^{2}$, RMSE and MAE computed on held-out data.

**Figure 6:**
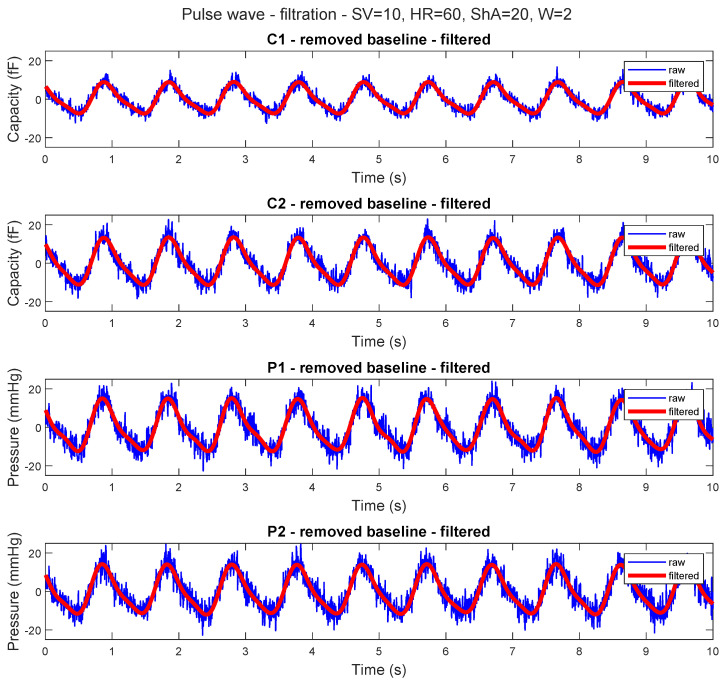
Comparison of the capacitive (volume pulse wave) and pressure sensor (pressure pulse wave) signals: raw values and after removal of the baseline and application of the low-pass filter.

Unlike in vivo conditions, our artificial arterial segments do not exhibit active vasodilation or vasoconstriction. Therefore, systemic vascular resistance is not dynamically regulated, and the variability in waveform shape is mainly driven by the controlled pump settings and the mechanical properties of the segments. The 15 artificial artery segments were connected separately for each measurement session, which takes at least 15 min to obtain enough pulse waves for further analysis. The acquired data were noisy, so filtration was needed (Figure 6).

For the next step, automatic detection of fiducial points was done using the conventional method proposed in the literature review [19,23,24]. The success rate of detections of the systole peak and foot was 99.93%, with a maximum error of one detection per measurement and sensor. For steepest point detection based on zero-crossing of the second derivative, the success rate was a little less than the systole peak and foot detector, that is, 99.84%. The dicrotic notch moment was detected using a non-conventional method, because the pulse wave shape on our vascular model differs from the real human body, and thus we used the alternative detection from the third derivative of the pulse wave as the first zero-crossing after the systole peak [34]. The efficiency of this detection was only 78.01% (for count of dicrotic notches over a 10 min signal). All identified fiducial points were double checked by a human operator; thus, we decided to continue with the systole peaks, feet, and steep points, and omit all dicrotic points, because they were not correctly detected.

The reduced detection accuracy of the dicrotic notch was mainly caused by its subtle and variable appearance in our waveforms and higher sensitivity of derivative-based detection to noise. Since the notch was not detected reliably, dicrotic features were excluded from further analysis to avoid propagating detection errors into the downstream models.

**Figure 7:**
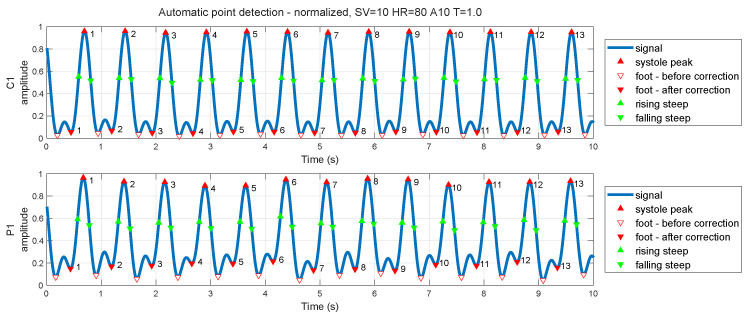
Corrected fiducial point detection on pulse wave.

Any inaccuracies in detection were corrected manually, but only for one measurement setup (stroke volume 10 mL, heart rate 80 bpm, Shore A hardness 10 and wall thickness 1 mm), where the backward pulse wave appears at the foot of the pulse wave in two sensors (C1 and P1). The correction was checked by three experts. The results of corrections—before and after—are shown in Figure 7.

**Figure 8:**
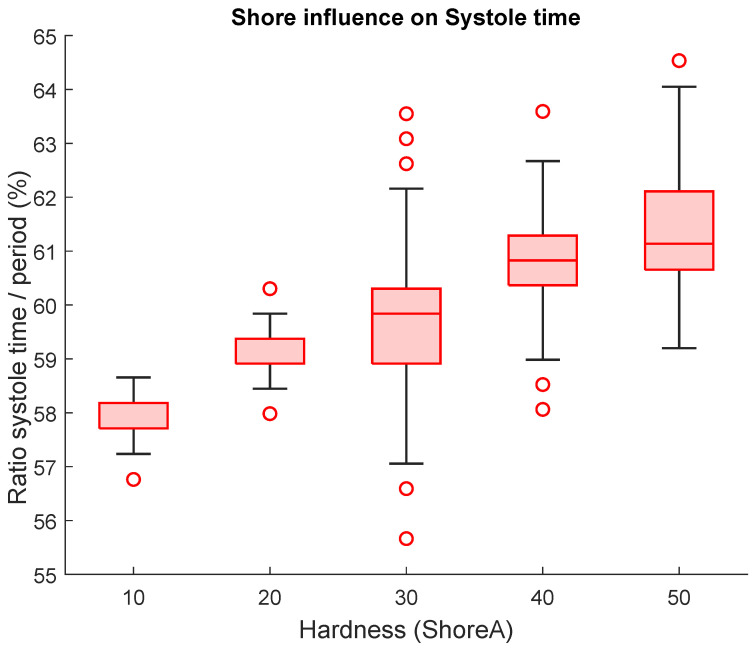
Systole time dependency on artificial artery hardness (HR = 60 bpm, SV = 20 mL, W = 2 mm).

For further analysis of time features (crest time and systole time), we normalized the measured durations using the corresponding cardiac cycle length derived from the measured heart rate (i.e., expressed as a percentage of the cycle). This normalization compensates for unavoidable heart rate variability across measurement setups and enables direct comparison between experiments. This step was taken to ensure that the time features were adjusted for variations in heart rate, allowing for more accurate and reliable comparisons between different measurement setups. Since the vascular model operates with continuous settings, it is not feasible to set the same heart rate for all measurements. Normalization was done as a percentage of the absolute feature time per measured cycle. Following this normalization, we were able to make direct comparisons between all the measurements. Our analysis revealed that higher Shore values were associated with longer systole times, suggesting that Shore hardness is a significant factor in our model, as supported by the boxplot analysis shown in Figure 8.

Figure 8 illustrates how the hardness of the silicon material used affects the shape of the pulse wave, as measured by the normalized systole time and its variance, using a heart rate of 60 bpm, stroke volume of 20 mL, and wall thickness of 2 mm.

Before fitting the linear regression model, outliers were identified using Cook’s distance. As a result, 7 measurements were removed from a total of 90 for the regression analysis, because they had disproportionate influence on the fitted coefficients.

### 3.1. Linear Regression

Multiple linear regression was used to test if our detected features (systole time (ST), pulse pressure (PP)) and directly measurable parameters such as stroke volume (SV), heart rate (HR) and wall thickness (W) predicted vascular hardness (ShA). The assumptions of linear regression were checked. The fitted regression model was as follows (Equation (2)):

(2) ShA=42.5−24.8ST+1.1PP−1.8SV−0.1HR−7.9W

The overall regression was statistically significant ($R^{2}$ = 0.73, *p* < 0.001).

It was found that all parameters predicted the hardness value (Table 3).

### 3.2. Adaptive Neuro-Fuzzy Inference System

The adaptive neuro-fuzzy inference system was trained on the measured dataset using 75% of the samples for training. The resulting system comprised 64 fuzzy rules (161 nodes) with 484 total parameters (448 linear and 36 nonlinear).

**Figure 9:**
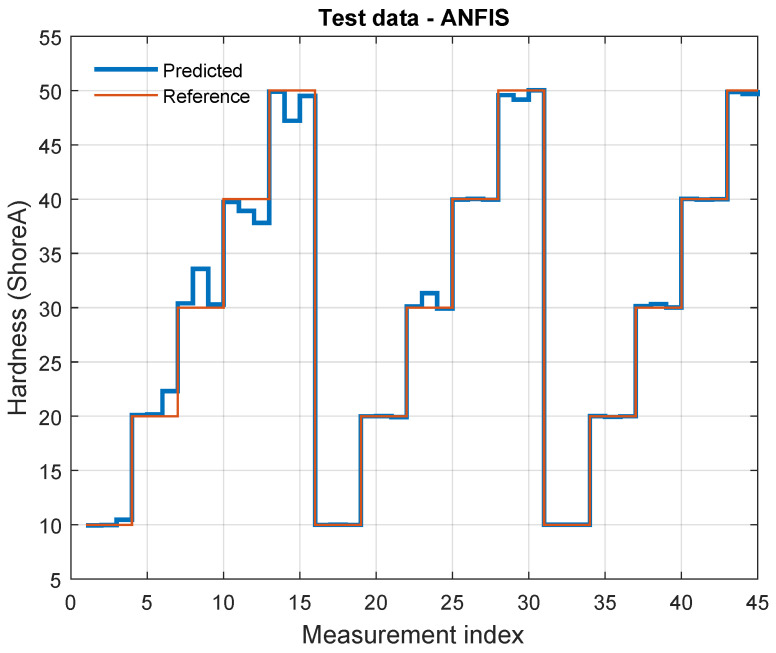
Comparison of predicted and actual Shore A hardness values. The reference values are discrete (10–50), reflecting the known material settings. The plot shows how accurately the ANFIS model predicts these values and highlights deviations from the ground truth.

The remaining 25% of the dataset were reserved for validation. Model performance was evaluated by comparing the predicted Shore A levels with the reference labels on this held-out subset (Figure 9).

The ANFIS model achieved $R^{2}=0.987$ on the held-out validation set within this in vitro dataset. In addition, the validation-set errors were RMSE = 0.701 and MAE = 0.250 Shore A units.

Table 4 provides a direct quantitative comparison between the ANFIS model and a transparent linear regression baseline using identical evaluation data. Error metrics were computed as mean absolute error (MAE) and root mean squared error (RMSE) between predicted and reference Shore A labels. The regression baseline was evaluated after excluding seven outlier observations to match the fitting protocol used for the reported regression equation. In addition to the ANFIS vs. linear regression comparison (Table 4), we report a broader benchmark against contemporary ML/DL baselines to provide a state-of-the-art context beyond simple linear models.

### 3.3. Benchmark Comparison with Contemporary ML and DL Models for Hardness-Level Prediction

To address the limitation of comparing ANFIS only against linear regression, we additionally evaluated a set of contemporary machine learning and deep learning baselines on the same feature set. The task was formulated as hardness-level prediction (discrete Shore A classes). Performance is reported using macro-averaged F1-score, together with macro sensitivity and specificity, which is more informative than accuracy in a small multi-class setting. All results are based on stratified 5-fold cross-validation with out-of-fold predictions, so each sample is evaluated only in a fold where it was not used for training. The reported metrics therefore reflect cross-validated performance on the available dataset. The feature set labeled FE corresponds to the engineered feature set described in the Materials and Methods section. Interestingly, the strongest baselines in both scenarios were polynomial regression models with regularization and snapping to the nearest discrete Shore A class. This suggests that a smooth continuous latent relationship between the features and hardness exists in the current dataset, while the final evaluation benefits from enforcing the discrete material grid.

Table 5 and Table 6 summarize the benchmark performance of contemporary machine learning and deep learning baselines for discrete Shore A classification. In the LOW_10_30 scenario (three classes), polynomial linear models achieved the highest macro-F1 and low prediction errors (MAE and RMSE). In the FULL_10_50 scenario (five classes), all baselines showed a marked performance decrease, indicating increased ambiguity between stiffness levels and a more challenging generalization setting. Importantly, reporting MAE and RMSE complements classification metrics by quantifying the magnitude of prediction errors in physically interpretable units.

In the reduced LOW_10_30 scenario (three hardness levels), performance increased substantially, with polynomial Lasso snapping reaching F1_*macro*_ = 0.889 (Sens_*macro*_ = 0.889, Spec_*macro*_ = 0.944). This improvement is expected due to reduced class overlap and fewer decision boundaries. Across both scenarios, regression-to-class approaches (continuous prediction followed by snapping to the nearest discrete Shore A level) consistently ranked among the strongest baselines, suggesting that the relationship between the engineered features and hardness is smooth but benefits from enforcing the discrete material grid in the final decision.

Inspection of the regularized polynomial models indicated that stiffness/compliance proxies based on the pulse pressure to stroke volume relationship (e.g., PP/SV and related interactions with wall thickness) were repeatedly selected among the highest-weight terms, which is consistent with the expected physical dependence between arterial stiffness and pressure–volume dynamics. All benchmark metrics were computed from out-of-fold predictions obtained by stratified 5-fold cross-validation, ensuring that each sample was evaluated only on a fold where it was not used for training.

### 3.4. Hardness to Elasticity Conversion

Variability in Shore A hardness and wall thickness influenced the pulse wave shape and, consequently, the PWV estimates. The learned models capture these relationships within the current dataset; however, the conversion from hardness to elasticity relies on empirical models that introduce additional uncertainty. While ANFIS handles nonlinear relationships effectively, the regression model is limited in this regard, as indicated by its lower R^2^. To further improve accuracy, future models could incorporate physically derived nonlinear elasticity functions or be trained on more finely graded material properties.

Based on the literature review, we found five empirical models (Table 1) for conversion of the silicone hardness material parameter to Young’s modulus. We performed calculations using these models to determine Young’s modulus values of the artificial arteries that we used in our physical vascular model. The five empirical models (Table 7) were used to convert Shore A hardness to Young’s modulus for silicone-like materials. The resulting elasticity values span a broad range across hardness levels and conversion formulas, which illustrates the non-negligible model-to-model variability introduced by this step.

The elasticity values in Table 7 were subsequently used as inputs for PWV calculations and for comparing the consistency of different conversion models against the measured PWV reference.

To validate the five Shore A to Young’s modulus conversion models, we compared their output against the measured PWV values derived from the physical setup. A comparative analysis revealed that the Ruess model consistently produced elasticity values that aligned most closely with the measured PWV using the Moens–Korteweg equation. Although no external mechanical testing was performed in this study, the chosen models were based on widely accepted empirical and theoretical relationships from the polymer mechanics literature. Future work should include benchmark validation using standardized test specimens and mechanical load experiments to further substantiate the selected conversion approaches.

### 3.5. Pulse Wave Velocity Calculation

The pulse wave velocity was calculated based on Moens–Korteweg Equation (1) using converted values of our arterial segment hardness shown in Table 8 for each ShA to elasticity conversion; we also obtained the real and observable values as in the human body (5–20 m/s) [8]. The increasing PWV values over 20 m/s could be consecutive of pathologies [8], which we can also simulate using stiffer arterial segments.

To verify the accuracy of PWV estimation using the above approaches, we used a dataset measured with a sampling frequency of 2000 Hz, as we were not able to use such a frequency due to the limitations of capacitive sensing. New validation measurements were performed, under the same settings as the vascular model.

Regarding the quality of estimations, described by the error (difference) of $PWV_{R}-PWV_{T}$ and $PWV_{F}-PWV_{T}$, we expected the values to be close to zero. Firstly, values were statistically tested for normal distribution using Shapiro–Wilk normality test with a 95% confidence level, which was rejected. Thus, for further verification we used a two-sided Wilcoxon test (Table 9) to evaluate whether the median estimation error differed from zero.

The results showed that the median estimation errors for each conversion model were not statistically different from zero at the 95% confidence level.

**Figure 10:**
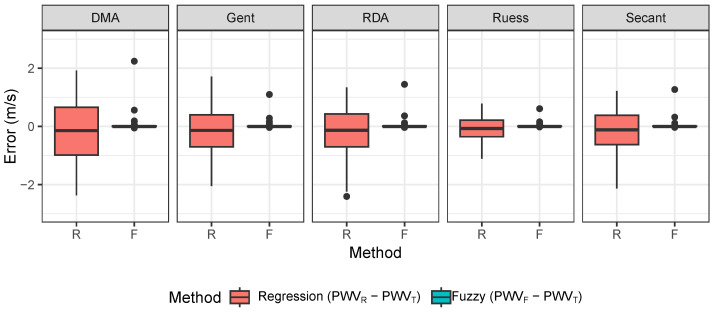
Error distribution across estimation methods.

Among the evaluated models, the Ruess conversion showed the smallest interquartile range (IQR) in the error distribution (Figure 10).

Although a formal variance-based sensitivity analysis was not applied in this study, the relative influence of input parameters was assessed indirectly by analyzing changes in PWV in response to variations in Shore A hardness, wall thickness, stroke volume, and heart rate. This parametric evaluation provided insight into which variables most significantly influenced the model’s output. Preliminary results suggest that Shore A hardness and wall thickness have a dominant effect on PWV estimation, aligning with theoretical expectations.

For further verification we used the $PWV_{T}$ values in comparison with the $PWV_{M}$. Normality based on Shapiro–Wilk’s test was not rejected, and thus Student’s *t*-test was used to test if mean values of error were significantly different from 0 (Table 10).

The Ruess model was selected as the most reliable based on its agreement with the measured PWV values within our experimental setup. While direct mechanical testing was not conducted, this selection is supported by the empirical performance observed in the in vitro validation. Further mechanical validation is planned for future studies.

**Figure 11:**
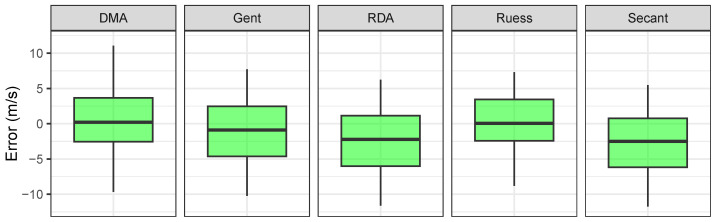
Differences between theoretical and measured PWV per model.

Based on statistical testing and Figure 11, we assumed that DMA, Gent and Ruess conversion models showed more acceptable results than the other two models.

Further analysis was performed on the difference between the values of $PWV_{R}$ and $PWV_{F}$ and $PWV_{M}$ on arterial segments for all 90 combinations of the measurement setup. Based on the Shapiro–Wilk test, the null hypothesis about normality was not rejected, thus we used Student’s *t*-test for testing that the mean value for each method and conversion model was equal to zero.

**Figure 12:**
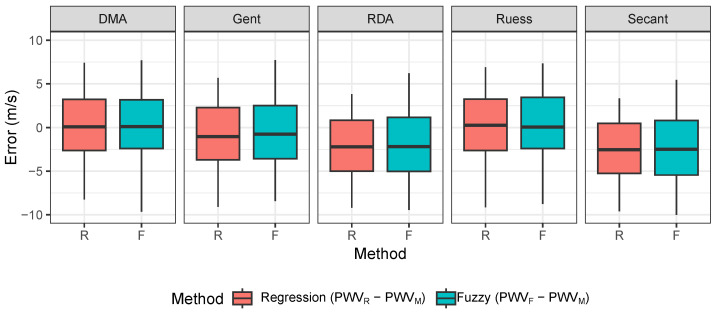
Comparison of PWV estimation methods for each conversion model.

In Table 11 we highlight the *p*-values greater than the significance level, thus we assume that the DMA, Gent and Ruess conversion models are not statistically significant for the difference from measured PWV. These results are also shown in Figure 12, where the medians of the mentioned conversion models are closest to 0. The reported *p*-values indicate whether the differences between the estimated and measured PWV values are statistically distinguishable from zero for each method and conversion model. In this dataset, the results suggest comparable agreement for selected conversion models (notably DMA, Gent, and Ruess), while other conversion models show systematic deviations.

However, in comparison to Figure 10, the IQR of error is higher between the estimated PWV and the measured PWV.

Based on our analysis of the methods and conversion models used, we found that the Ruess conversion model was better suited for our analysis of pulse wave velocity estimated using pulse wave features and other input parameters describing our measurement setup of the physical vascular model.

## 4. Discussion

Experimentation on artificial arterial segments provides a controlled and reproducible framework for early-stage method development and validation of stiffness-related inference and PWV derivation in vitro. The artificial segments were manufactured with different materials and wall thicknesses to span a simplified range of mechanical conditions, and subsequently tested on our physical vascular model under defined boundary conditions.

The selection of three wall thicknesses and five Shore A hardness levels was made to cover a practical range of arterial stiffness conditions while maintaining experimental feasibility and repeatability. These settings should be interpreted as simplified mechanical conditions rather than direct physiological equivalents of specific patient groups. The limitations in range were also influenced by material availability and the mechanical constraints of the silicone tubes used. Although this does not fully capture the diversity of human vascular conditions, it provides a controlled starting point for evaluating model sensitivity. Future studies will incorporate more finely graded variations and broader biological relevance.

To evaluate these artificial arterial segments, a range of tunable parameters were selected on our vascular model. The pulse waveform was then captured using our contactless capacitive sensor and a gold standard invasive pressure sensor. The invasive pressure sensor served as a reference measurement, enabling a direct comparison of waveform-derived features extracted from both sensing modalities under identical conditions. In addition, we were able to maintain a constant value of the mean blood pressure, thus mimicking the baroreflex function using an electrically controllable valve. This last element controls systemic vascular resistance by reducing the flow of the liquid. With a pressure maintained at 30 mmHg, we were therefore able to analyze the pulse pressure of both sensors under identical conditions.

Experimental results showed that our consideration of the artificial arterial segments and their material hardness was significant for elasticity measurement. Measurement was performed in 90 different settings twice, firstly with a sampling frequency of 200 samples per second for both types of sensors, and secondly with a validation measurement with a much higher sampling frequency of 2000 sps, to be able to measure the pulse wave velocity on such short arterial segments. This first measurement was sufficient for the prediction models of pulse wave features based on our tunable parameters from our physical vascular model. The R-squared coefficients of determination for these models were 66% for crest time, 81% for systole time and 35% for pulse pressure. The observed increase in systolic duration with higher Shore A hardness suggests that stiffer arterial walls slow down the propagation and relaxation of the pressure wave. This mimics real physiological behavior, where increased arterial stiffness—common in aging or hypertension—is associated with altered pulse wave dynamics and prolonged systolic phases.

Based on these predictions, we performed an inverse mapping step to estimate the Shore A hardness of the artificial arterial wall from waveform-derived features. For Shore A hardness estimation, we evaluated two approaches: (i) linear regression as an interpretable baseline and (ii) an adaptive neuro-fuzzy inference system (ANFIS) as a nonlinear fuzzy-learning model. The regression model used systolic time, pulse pressure, stroke volume, heart rate, and wall thickness as predictors and achieved an $R^{2}$ of 0.73. In contrast, ANFIS achieved substantially higher agreement with the reference Shore A labels (Table 4), indicating improved ability to capture nonlinear dependencies between waveform morphology and effective stiffness in the in vitro setup.

Using ANFIS creates a more flexible and accurate prediction model compared to traditional linear regression. It combines fuzzy logic with neural learning, making the system more robust and better at adapting to different arterial conditions. This demonstrates the strength of computational intelligence in medical applications. The notable difference in R-squared values—98.7% for ANFIS and 73% for the regression model—reflects the ANFIS model’s ability to capture nonlinear and fuzzy relationships among input parameters. The regression model, while interpretable, assumes linearity, and thus fails to capture more complex dynamics. To avoid overfitting, the ANFIS model was trained on 75% of the data and validated on the remaining 25%, achieving consistently high performance. These results suggest that the improved accuracy is due to model capacity rather than overfitting.

In addition to the ANFIS vs. linear regression comparison, we benchmarked multiple contemporary ML/DL baselines under two classification granularities (LOW_10_30 vs. FULL_10_50; see Table 5 and Table 6). A consistent trend across all baselines is the performance gap between the LOW_10_30 and FULL_10_50 scenarios. Increasing the number of discrete hardness levels substantially increases class overlap and ambiguity, resulting in higher MAE/RMSE, even for the strongest polynomial and ordinal DL models. This indicates that Shore A inference from waveform-derived features becomes increasingly challenging as the stiffness scale is refined, supporting the need for nonlinear and hybrid approaches under more demanding settings. Although controlled in vitro conditions reduce physiological variability, the observed degradation of ML/DL baseline performance in the five-class setting demonstrates that the task is not trivial and remains sensitive to feature overlap and model capacity, motivating robust external validation as future work.

Because the mapping between Shore A hardness and Young’s modulus is empirical and not standardized across silicone materials, we treated the Shore A-to-elasticity conversion as an uncertainty and sensitivity factor. Specifically, five published conversion models were evaluated to quantify how variability in elasticity estimates propagates into derived PWV values. In addition, the manufacturer-reported Shore A tolerance (approximately ±2) represents a further source of uncertainty in the estimated modulus.

After conversion from Shore A hardness, the elasticity data were fed into the Moens–Korteweg Equation (1), commonly used for PWV estimation. We used directly all four input variables of the equation: the elasticity, wall thickness, blood density and arterial diameter.

We calculated pulse wave velocity using our arterial segments’ Shore A hardness values, which we labeled as theoretical PWV values. Then, we used elasticity values based on regression and fuzzy method estimation. Afterwards, we calculated the differences between the values of the methods used and the theoretical values ($PWV_{R}-PWV_{T}$ and $PWV_{F}-PWV_{T}$), and this error we plotted as boxplots for each elasticity conversion model. In addition to the boxplot we also performed a statistical analysis. All elasticity conversion model medians of errors were not statistically significant from 0. However, we concluded from the boxplots that the interquartile ranges were smallest for the Ruess model in the regression and fuzzy methods. To evaluate systematic errors across different PWV estimation methods, the results were compared against measured values ($PWV_{M}$) under controlled conditions. Theoretical PWV ($PWV_{T}$), calculated using the Moens–Korteweg equation, tended to overestimate values due to idealized biomechanical assumptions. Regression-based estimates ($PWV_{R}$) exhibited larger deviations, reflecting the limited capacity of linear models to capture nonlinearities in feature-to-stiffness mapping. In contrast, fuzzy inference estimates ($PWV_{F}$) showed reduced error magnitudes and improved agreement with $PWV_{M}$, indicating higher robustness in this controlled setting. The error distribution further suggested that $PWV_{T}$ errors were systematic, while model-based estimates showed additional variance linked to conversion uncertainty and overlapping material conditions.

For further analysis we used the real measured pulse wave velocity ($PWV_{M}$) on our 30 cm long artificial arterial segment and calculated the differences between all methods according to the $PWV_{M}$ values. We concluded from the statistical testing that the mean differences of all methods from the DMA, Gent and Ruess conversion models were not statistically significant from 0. But based on exploratory analysis, the smallest IQRs from boxplots were from the Ruess and DMA conversion models (IQR about 6 m/s). These differences between estimated and measured pulse wave velocity could be caused by accumulating error due to inaccuracies of the silicone material used, further inaccuracy from the regression or fuzzy method used for estimation, or error caused by the conversion models. These results suggest the feasibility of deriving PWV-related estimates from a single-site waveform in a controlled in vitro setting; however, translation to in vivo measurements will require validation under physiological variability, wave reflections, and measurement noise.

Due to the limitations of our model, which is unable to replicate the full complexity of the real vascular system, the shape of the pulse wave we obtained differs from that of a human pulse wave. As a result, we were unable to collect data on all of the features that could be detected in a real pulse wave; for example, the Large artery stiffness index—subject height divided by time between systolic and diastolic peak; the Augmentation index—ratio between amplitude of systolic and amplitude of diastolic peak; and many ratios of peaks from a, b, c, d and e waves from second derivative corresponding to arterial stiffness. Using all these mentioned features, the prediction model precision could probably be increased, but this must be included in further testing and analysis. Although the artificial artery setup provides high reproducibility and controlled conditions, it lacks several physiological complexities found in vivo. In vitro arterial phantoms are valuable for early-stage method development and controlled validation, as also demonstrated in prior PPG-based stiffness studies. However, clinical translation requires external validation under biological variability and realistic wave reflections [7,25]. Real arteries exhibit viscoelastic behavior and non-Newtonian blood flow and are subject to active vascular regulation, conditions which are not replicated in this setup. Furthermore, the silicone tubes used have uniform and isotropic properties, whereas biological tissues are heterogeneous and anisotropic. These factors may limit the generalizability of the results to clinical conditions. Future validation on ex vivo or in vivo models is necessary to fully assess real-world applicability.

Overall, the proposed workflow provides a reproducible in vitro benchmark for stiffness-related inference and PWV derivation under known boundary conditions, while clinical translation remains a subject of future validation.

The presented results were obtained in a controlled in vitro setup using artificial silicone vessels. Such segments do not replicate the full nonlinear, viscoelastic, and inhomogeneous behavior of living arteries, including pressure-dependent stiffness, wall anisotropy, endothelial effects, wave reflections, and physiological variability. Therefore, clinical conclusions are outside the scope of this study and require validation on biological tissues or in vivo recordings.

## 5. Conclusions

Creating artificial arterial segments with different materials and wall thicknesses to mimic the human vascular system is a big step forward in vascular research. Our study showed that the hardness of these artificial segments significantly affects the blood pressure pulse wave pattern, which allows us to accurately estimate pulse wave velocity (PWV) from just one measurement site. By using parameters from our vascular model, such as stroke volume, heart rate, wall thickness, and pulse wave features, we improved the traditional method for estimating PWV.

We found that both linear regression and adaptive neuro-fuzzy inference systems (ANFIS) can effectively estimate the hardness of the arterial segments, with ANFIS being more precise. Among the different models we tested for converting hardness to elasticity, the Ruess model was the most accurate, providing PWV values similar to those in real human arteries.

These findings should be interpreted as an in vitro feasibility and validation step rather than evidence of clinical applicability or superiority in real-world diagnostics. Translation to biological tissues or in vivo settings will require external validation, calibration of hardness-to-elasticity mapping against mechanical testing, and robustness analysis under physiological variability, reflections, and measurement noise.

While the ANFIS model demonstrated strong performance in this controlled setting, further validation in real-world and clinical environments is necessary to confirm its reliability and generalizability.

## Figures and Tables

**Table 1:** Conversion models from Shore to Young’s modulus for silicone material.

Model	Young Modulus (E)	Reference
DMA	$0.2354\times e^{0.0657ShA}$	[17]
Gent	$\frac{0.0981\left(53+7.62336ShA\right)}{0.137505\left(254-2.54ShA\right)}$	[18]
RDA	$0.1611\times e^{0.058ShA}$	[17]
Ruess	$e^{0.0235ShA-0.6403}$	[17]
Secant	$0.1614\times e^{0.0541ShA}$	[17]

**Table 2:** ANFIS configuration used in this study.

FIS type	Sugeno (first-order)
Inputs/Output	6/1
Membership functions	Gaussian (gaussmf)
Rule initialization	Data-driven (clustering-based)
Training algorithm	Hybrid (LSE + gradient-based updates)
Epochs	30
Defuzzification	Weighted average

**Table 3:** Regression model output for Shore A hardness estimation.

Hardness (ShA)	Estimate	Standard Error	t-Value	*p*-Value
(Intercept)	42.5	3.8	11.0	<0.001
ST	−24.8	8.5	−2.9	0.004
PP	1.1	0.1	15.3	<0.001
SV	−1.8	0.2	−10.8	<0.001
HR	−0.1	0.1	−2.8	0.007
W	−7.9	1.1	−7.1	<0.001

**Table 4:** Side-by-side quantitative comparison for Shore A hardness estimation.

Model	$R^{2}$	RMSE	MAE
Linear regression	0.732	7.266	5.499
ANFIS	0.987	0.701	0.250

**Table 5:** Performance comparison of contemporary ML/DL baselines for Shore A classification in the FULL_10_50 scenario (five classes, N = 90, feature set FE).

Model	F1 (Macro)	Sens.	Spec.	MAE (ShA)	RMSE (ShA)
ML_Poly_LassoSnap	0.610	0.600	0.900	4.444	7.746
ML_Poly_RidgeSnap	0.590	0.578	0.894	4.556	7.379
DL_Ordinal_CORAL	0.558	0.544	0.886	4.667	6.992
ML_RF_Reg_Snap	0.555	0.567	0.892	4.778	7.528
ML_HistGB_Reg_Snap	0.534	0.522	0.881	5.222	7.817
DL_MC_Dropout_MLP	0.545	0.556	0.889	5.556	8.819

**Table 6:** Performance comparison of contemporary ML/DL baselines for Shore A classification in the LOW_10_30 scenario (three classes, N = 54, feature set FE).

Model	F1 (Macro)	Sens.	Spec.	MAE (ShA)	RMSE (ShA)
ML_Poly_LassoSnap	0.889	0.889	0.944	1.296	4.082
ML_Poly_RidgeSnap	0.868	0.870	0.935	1.481	4.303
ML_RidgeSnap	0.838	0.833	0.917	1.667	4.082
DL_Ordinal_CORAL	0.833	0.833	0.917	1.852	4.714
DL_MC_Dropout_MLP	0.808	0.815	0.907	2.037	4.907
DL_MLP_softmax	0.795	0.796	0.898	2.222	5.092

**Table 7:** Conversion models from Hardness to Elasticity.

Hardness (Shore A)	Elasticity (MPa)				
	DMA	Gent	RDA	Ruess	Secant
10	0.45	0.41	0.29	0.67	0.28
20	0.88	0.73	0.51	0.84	0.48
30	1.69	1.14	0.92	1.07	0.82
40	3.26	1.69	1.64	1.35	1.41
50	6.29	2.46	2.93	1.71	2.41

**Table 8:** Theoretical PWV ($PWV_{T}$) calculated from material hardness.

Wall Thickness (mm)	Hardness (Shore A)	${\mathrm{PWV}}_{T}$ (m/s)				
		DMA	Gent	RDA	Ruess	Secant
1	10	6.74	6.42	5.36	8.17	5.27
	20	9.36	8.56	7.17	9.18	6.90
	30	13.00	10.69	9.58	10.33	9.04
	40	18.05	13.00	12.80	11.62	11.85
	50	25.07	15.67	17.11	13.06	15.54
1.5	10	8.25	7.87	6.57	10.00	6.45
	20	11.46	10.48	8.78	11.25	8.45
	30	15.92	13.09	11.73	12.65	11.08
	40	22.11	15.92	15.68	14.23	14.52
	50	30.71	19.19	20.96	16.00	19.03
2	10	9.53	9.08	7.59	11.55	7.45
	20	13.24	12.10	10.14	12.99	9.76
	30	18.38	15.12	13.55	14.61	12.79
	40	25.53	18.38	18.11	16.43	16.76
	50	35.46	22.16	24.20	18.48	21.97

**Table 9:** Wilcoxon test results: $PWV_{R}$, $PWV_{F}$ vs. $PWV_{T}$.

Method	Wilcoxon	DMA	Gent	RDA	Ruess	Secant
Regression	V	285	291	285	285	286
	*p*-value	0.323	0.370	0.323	0.323	0.331
Fuzzy	V	426	426	425	427	425
	*p*-value	0.268	0.268	0.274	0.261	0.274

**Table 10:** Student’s *t*-test: $PWV_{T}$ vs. measured $PWV_{M}$.

*t*-Test	DMA	Gent	RDA	Ruess	Secant
*t*	0.276	−1.504	−3.473	0.100	−3.974
*p*-value	0.784	0.141	0.001	0.921	<0.001

**Table 11:** Student’s *t*-test: $PWV_{R}$, $PWV_{F}$ vs. $PWV_{M}$.

Method	*t*-Test	DMA	Gent	RDA	Ruess	Secant
Regression	*t*	−0.140	−1.908	−4.122	−0.018	−4.579
	*p*-value	0.890	0.064	<0.001	0.986	<0.001
Fuzzy	*t*	0.393	−1.452	−3.425	0.137	−3.940
	*p*-value	0.696	0.155	0.002	0.892	<0.001

## Data Availability

Daniel Barvik 2023 PWV capacity pressure. Available at https://doi.org/10.21227/8d1r-b939.
